# Modulation of Epithelial Mesenchymal Transition after AGTR-1 Gene Edition by Crispr/Cas9 and Losartan Treatment in Mammary Tumor Cell Line: A Comparative Study between Human and Canine Species

**DOI:** 10.3390/life11121427

**Published:** 2021-12-18

**Authors:** Marina Gobbe Moschetta-Pinheiro, Jucimara Colombo, Bianca Lara Venâncio de Godoy, Julia Ferreira Balan, Bianca Carlos Nascimento, Debora Aparecida Pires de Campos Zuccari

**Affiliations:** 1 PostGraduate Program in Health Sciences, Faculdade de Medicina de São José do Rio Preto (FAMERP), Avenida Brigadeiro Faria Lima, 5416, São José do Rio Preto 15090-000, Brazil; bianca.godoy@edu.famerp.br; 2 Department of Health Sciences, Universidade Paulista (UNIP), Avenida Juscelino K. de Oliveira, s/n, São José do Rio Preto 15091-450, Brazil; 3 Laboratório de Investigação Molecular no Câncer (LIMC), Faculdade de Medicina de São José do Rio Preto (FAMERP), Avenida Brigadeiro Faria Lima, 5416, São José do Rio Preto 15090-000, Brazil; jucimara.colombo@famerp.br (J.C.); julia.balan@edu.famerp.br (J.F.B.); bianca.nascimento@edu.famerp.br (B.C.N.); debora.zuccari@famerp.br (D.A.P.d.C.Z.); 4 PostGraduate Program in Genetics, Instituto de Biociências, Letras e Ciências Exatas (UNESP/IBILCE), Rua Cristovão Colombo, 2265, São José do Rio Preto 15054-000, Brazil

**Keywords:** AGTR-1, gene edition, losartan, mammary tumors, triple negative cell lines

## Abstract

Breast cancer is the most prevalent tumor type among women and female dogs. Tumor malignancy is characterized by the epithelial-to-mesenchymal transition (EMT) which leads to the metastasis formation. The inhibition of angiotensin II type I receptor (AGTR1) by an antagonist such as losartan can suppress angiogenesis, consequently contributing to the metastasis control. The aim of this study was to analyze the capacity of losartan and AGTR-1 gene edition to modulate the EMT process in triple negative/metastatic mammary tumor cells, compared to existing treatment protocols such as carboplatin. The cell lines CF41.Mg and MDA-MB-468, were cultured and treated with carboplatin, losartan, or submitted to AGTR-1 gene edition by CRISPR/Cas9. EMT markers and PARP-1 protein and gene expression were evaluated by immunofluorescence or immunocytochemistry and qRT-PCR, respectively. Cell migration capacity was also evaluated. For CF41.Mg and MDA-MB-468 cell lines, there was an increase in E-cadherin and a decrease in N-cadherin and PARP-1 protein and gene expression after treatment with carboplatin, losartan, both in combination and after AGTR-1 gene edition. There was a decrease in VEGF and PARP-1 protein and gene expression after AGTR-1 gene edition. Moreover, in both lines, reduction in invasion rate was observed after all treatments. Our data suggest that losartan and the gene edition of AGTR-1 by CRISPR/Cas9 were able to block the DNA repair and control the EMT process, such as carboplatin. The results in the canine species are unprecedented, as there are no data in the literature that demonstrate the action of losartan in this tumor type.

## 1. Introduction

Breast cancer (BC) is the most frequent type of cancer among women; this type of tumor ranks second among causes for cancer related death in women. In addition, the incidence and mortality of this neoplasm should significantly increase in the next years [1].

Several genetic and environmental factors, especially those co-existing, increase the risk of occurrence, morbidity, and recurrence of BC. Environmental and lifestyle factors include ionizing radiation, hormone therapy, and alcohol, as well as dietary and socioeconomic factors, obesity, menopause, non-breastfeeding, and lack of physical activity. Other well-recognized and documented risk factors include age and familial genetic predisposition [2,3].

Similar to humans, breast tumors are described as the most frequent neoplasm in female dogs. Dogs develop cancer spontaneously with age, with genetic differences related to race-specific risk. The high prevalence in some breeds suggests a genetic component, as it occurs in familial human breast cancer [4]. Unfortunately, the most effective treatment in breast cancer is surgical excision, however, due to the high incidence of metastases in this type of tumor, surgery alone is not efficient in all canine patients [5].

All breast tumor diagnosis begins with classification based on the detection of estrogen (ER), progesterone (PgR), and human epidermal growth factor-2 (HER2) receptors by immunohistochemistry (IHC) combined with molecular and gene expression analyses [3]. Triple negative breast cancer (TNBC) is characterized by low expression of ER and PgR as well as HER2. Thus, this type of cancer does not respond to target or hormonal therapies, with limited treatment options, contributing to a worse prognosis [3,6].

The classification system of canine mammary tumors into subtypes luminal A, luminal B, HER2-positive, and triple negative, based on the expression of tumor markers: estrogen receptor, progesterone receptor, and HER2, it is not common in canine species. However, in a study by our group, we perform this classification in 110 canine breast tumors and we observed that luminal A and B tumors were associated with better prognosis, while triple negative and HER-2 positive tumors were more aggressive and were associated with the occurrence of metastasis, worse TNM classification, and shorter survival time. Thus, we demonstrate through our work the importance of canine breast tumor classification. Moreover, the triple negative canine tumor also has an aggressive behavior in female dogs [7].

Treatment for BC includes surgery, hormone therapy, radiation, and chemotherapy. In both species, carboplatin is one of the compounds used in the treatment of breast cancer, both in the early or advanced stages of the disease. However, chemotherapy can increase intratumoral heterogeneity due to limitations in tissue permeability and variable concentrations of drug exposure in tumor cells. Tumor cells close to the blood vessels undergo apoptosis due to exposure to high concentrations of the drugs, while those at a moderate distance undergo senescence. Tumor cells located far from the vessels are not sensitive to chemotherapy drugs and can become foci of tumor recurrence [8].

About 30% of patients with early stage breast cancer will progress to severe forms of the disease, developing distant organ metastases. An invasive phenotype of tumors is mediated by a molecular mechanism called epithelial–mesenchymal transition (EMT). This molecular mechanism is strongly related to cell migration and invasion and consequently to the metastatic process [9,10].

EMT is a biological process in which epithelial cells show low expression of proteins involved in cell junctions, such as E-cadherin, and obtain a mesenchymal phenotype, which is observed by the high expression of mesenchymal markers, such as N-cadherin and vimentin. The activation of EMT plays an important role in the initial stages of the metastatic cascade, increasing the migratory and invasive abilities of tumor cells. Thus, blocking the EMT process may represent an interesting approach to inhibit the migration and invasion process of TNBC cells [11].

Despite the progress made in the field of screening, diagnosis, and therapeutic strategies in the management of breast cancer, the poor prognosis in TNBC and drug resistance have important limitations, which are also current challenges to contain the disease [1].

The angiotensin II type I receptor (AGTR1) is a protein component of the cell membrane. This receptor is part of the group of G protein-coupled receptors, and when it binds to angiotensin II, it causes several cellular reactions to increase blood pressure, one of the most important being vasoconstriction. AGTR1 has shown the potential to stimulate cell growth, migration, and invasion, as well as to promote angiogenesis, inflammation, immune response, and lymph node metastasis [12,13].

In turn, losartan is a drug in the class of angiotensin receptor antagonists. Its main indication is the treatment of high blood pressure [14]. Thereby, the inhibition of AGTR1 by antagonists such as losartan can inhibit angiogenesis, thus contributing to the suppression of tumor growth and the occurrence of metastases. Nevertheless, the role of AGTR1 in breast cancer lymph node metastasis is still poorly understood [13]. As demonstrated by the study of Li et al. (2021) [15], the administration of losartan improved the outcome of radiotherapy and decrease metastases in murine BC model.

In this context, the aim of this study was to analyze the capacity of losartan treatment and AGTR-1 gene edition to modulate the EMT process in triple negative/metastatic canine and human mammary tumor cells.

## 2. Materials and Methods

### 2.1. Cell Line Validation Statement

The CF41.Mg cell line was previously characterized by our research group [16] as a triple negative and metastatic canine mammary tumor cell line, while the metastatic human tumor cell line MDA-MB-468 was purchased from American Type Culture Collection (ATCC, Manassas, VA, USA).

### 2.2. Cell Culture Procedure

The cells were cultured inside an incubator, at 37 °C and 5% CO_2_, in a specific culture flask (75 cm^2^) (Sarstedt, Nümbrecht, Germany) containing DMEM culture medium with a high concentration of glucose (Cultilab, São Paulo, Brazil) and supplemented with 10% fetal bovine serum (FBS, Cultilab, São Paulo, Brazil) and 1% of penicillin (100 IU/mL) and streptomycin (100 μg/mL) (Sigma-Aldrich, St. Louis, MO, USA). To stimulate cell growth, the culture medium was changed every two days. When the cells reached 80 to 100% confluence, they were detached by the addition of trypsin-ethylenediaminetetraacetic acid solution (EDTA) (Sigma-Aldrich, St. Louis, MO, USA) and counted with a 4% trypan blue solution in a Neubauer chamber to check the number of viable cells. To carry out the other experiments, the cells were transferred to culture flasks specific to each technique.

### 2.3. Protein Expression by Immunofluorescence Assay

To analyze the protein expression of EMT markers and PARP-1 was performed the immunofluorescence assay according to Gelaleti et al. (2017) [17]. Briefly, 6 × 10^4^ cells of CF41 and MDA-MB-468 cell lines were transferred to a silicone separator attached to a slide and kept in the incubator for 24 h at 37 °C with 5% CO_2_. Then, the cells were treated with carboplatin, losartan, or both in combination. For the immunofluorescence technique were used the specifics primary antibodies E-cadherin (1:200) (catalogue number 3195S, Cell Signaling Technology, Danvers, MA, USA), N-cadherin (1:100) (catalogue 7939, Santa Cruz Biotechnology, Dallas, TX, USA) and PARP-1 (1:50) (catalogue 436400, Thermo Fisher Scientific, Waltham, MA, USA) and the secondary antibody used was Alexa Fluor 648 anti-mouse IgG (1:100) (Sigma-Aldrich). To analyze the protein expression, the cells were captured using a microscope containing a specific software (OLYMPUS, model BX53, software Image-Pro Plus version 7.0, Rockville, MD, USA). All experiments were carried out in triplicate and protein expression was quantified according to Jardim-Perassi et al. (2014) [18] as cited by Gelaleti et al. (2017) [17]. The analyses were performed using ImageJ Software (NIH, Bethesda, MD, USA) and all values were obtained in arbitrary units (a.u.) and represented as the mean optical density (M.O.D.).

### 2.4. Protein Expression by Immunocytochemistry Staining

The cells were plated (6 × 10^4^ cells) and submitted to gene edition of AGTR-1 by CRISPR/Cas9. After, cells were fixed in 4.0% paraformaldehyde and the primary antibodies were used to delineate the expression of corresponding antigens: VEGF (1:300) (Santa Cruz Biotechnology, Dallas, TX, USA) and PARP-1 (1:50) (catalogue 436400, Thermo Fisher Scientific) The immunocytochemistry staining procedures were performed using Reveal System of Detection without Biotin Kit (Biogen, Cambridge, MA, USA) according to the manufacturer’s instructions. Briefly, cells were washed with distilled water and incubated with citrate buffer at 96 °C for 35 min for antigen retrieval. Once it was washed with PBS the sections were incubated with 0.1% of hydrogen peroxide for 15 min and protein blocking for 10 min. Following this process, sections were incubated with the primary antibody diluted in PBS/albumin 5% at 4 °C overnight and with secondary antibody (anti-mouse and -rabbit immunoglobulins). The sections were washed once again and incubated with HRP peroxidase conjugate followed by chromogenic substrate (3,3′-diaminobenzidine tetrahydrochloride—DAB) (Biogen, Cambridge, MA, USA). At the end, all sections were counter stained with hematoxylin, dehydrated and cover slipped with Erv Mount (Easypath, Sao Paulo, Brazil).

### 2.5. Gene Expression by Quantitative Real-Time RT-PCR (qRT-PCR)

The expression of E-cadherin, N-cadherin, PARP-1, and VEGF gene was determined by qRT-PCR. Approximately 1 × 10^6^ cells of the canine mammary tumor cell line CF41.Mg and human MDA-MB-468 were transferred to 5 wells culture plates. Then, the cells were treated with carboplatin, losartan, or both in combination, or submitted to gene edition of AGTR-1 by CRISPR/Cas9. The Trizol reagent (Sigma Aldrich, St. Louis, MO, USA) was used to extract the total RNA and its concentration was determined using a NanoDrop 2000 Spectrophotometer (Thermo Fisher Scientific). To obtain the cDNA the reverse transcriptase-poly20merase chain reaction (RT-PCR) was developed using the High Capacity cDNA Kit (Applied Biosystems, Foster City, CA, USA). A qRT-PCR reaction was performed in triplicate on StepOnePlus Real Time PCR system (Applied Biosystems, Foster City, CA, USA) using TaqMan Universal Master Mix (Applied Biosystems, Foster City, CA, USA). Taqman inventoried assays used for the canine species were: VEGF (Cf02623449_m1), PARP-1 (Cf02630973_m1), and the sequences used for endogenous control was previously described by Moschetta et al. (2019) [19]. For human species, the inventoried assays used were VEGF (Hs00900055_m1), PARP-1 (Hs00242302_m1), and endogenous controls were GAPDH (Hs99999905_m1) e ACTB (Hs99999903_m1) (Applied Biosystems, Foster City, CA, USA). Each cDNA sample was at a concentration of 100 ng. The amplification was performed in cycles at 95 °C for 10 min, followed by 40 cycles at 95 °C for 15 s and 60 °C for 1 min. The relative quantification (RQ) value of the expression of interest genes was determined with DataAssist 3.0 software (Applied Biosystems, Foster City, CA, USA), using the average of the normalizing genes used as endogenous control (ΔΔCt) [20].

### 2.6. Migration Assay

Initially, 1 × 10^6^ cells/well of 6 wells plates were treated with carboplatin, losartan, both in combination or submitted to AGTR-1 gene edition by CRISPR/Cas9. At the end of 24 h of treatment, the cells were detached and harvested using trypsin-ethylenediaminetetraacetic acid solution (EDTA) (Sigma-Aldrich, St. Louis, MO, USA), counted in a Neubauer chamber with a 4% trypan blue solution to check the number of viable cells, and then transferred to 24 well plates containing 8 μm inserts BDMatrigel*™* membranes (Becton Dickinson Labware^®^, Billerica, MA, USA). Following the manufacturer’s instructions, 0.5 mL of culture medium enriched with 10% FBS (chemoattractant) was added to the bottom of the plate wells, while inside the inserts containing the matrigel membrane was added 2 × 10^4^ cells previously treated in 0.3 mL of culture medium without FBS. After 24 h of incubation, these membranes had been washed with PBS 1x and fixed with 4% formaldehyde for 20 min at room temperature. The impermeabilization of the cells was then performed with 100% methanol for 20 min, at room temperature, followed by staining with hematoxylin for 5 min to detect migratory cells. The cell migration rate analysis was performed according to Galeti et al. (2021) [21].

### 2.7. AGTR-1 Gene Edition by CRISPR/Cas9

For knockout of AGTR-1, the genomic edition will be mediated by TrueGuide™ Synthetic gRNA (catalogue A35513, Thermo Fisher Scientific) AGTR1 Human (ID: GRFVK2T) and AGTR1 Canine (ID: GRDJXWX) using Lipofectamine™ CRISPRMAX™ reagent (Thermo Fisher Scientific) for transfection. The CRISPR/Cas9 was performed according to the manufacturer’s instructions (Invitrogen™ TrueGuide™ Synthetic gRNA). 

The cells were plated (Table 1) and after 24 h the transfection was performed by Lipofectamine™, which is a lipid nanoparticle transfection reagent for CRISPR-Cas9 protein delivery. For the preparation of the transfection complex (Table 1), first Tube 1 was prepared containing: Cas9 protein + sgRNA Solution with Cas9 Plus™ Reagent in Opti-MEM™ I Medium. Then, Tube 2 was prepared by diluting Lipofectamine™ CRISPRMAX™ reagent in Opti-MEM™ I Medium. Tube 2 was incubated for 1 min at room temperature. After, the Tube 2 was added to the Tube 1 and it was mixed by pipetting. This mixture was incubated for 10 min at room temperature, followed by the addition of this transfection complex (10 μL in each well of 96 wells plate, 50 μL in 24 wells and 250 μL in 6 wells) to the previously plated cells and then incubated at 37 °C. After 2 days of incubation, the culture medium was removed and the adherent cells were washed with PBS to proceed with the other applications. 

### 2.8. Statistical Analysis

The results were previously submitted to descriptive analysis to determine statistical normality. For samples with normal distribution, Analysis of Variance (ANOVA) was used, followed by the Bonferroni or Dunnett’s Multiple Comparison test and for those with non-normal distribution, the Kruskal–Wallis test was used. All values obtained were expressed as mean ± standard deviation (S.E.M.). Values of *p* < 0.05 were considered significant. All analyses were performed using GraphPad Prism9 software.

## 3. Results

### 3.1. Losartan Treatment Decreased EMT Markers Protein Expression in Both Tumor Cell Lines

The platinum agents are chosen as the first treatment option in combination with other older pharmacological agents and, despite the side effects, patients with TNBC respond well to treatment with these compounds. It is not clear the mechanism of action of platinum agents, but deoxyribonucleic acid (DNA) adducts are formed [22]; in this way we added losartan to the treatment to control the generation of EMT process and compared its action with the results achieved after treatment with carboplatin, which is the compound used worldwide for the treatment of breast cancer.

**Figure 1:**
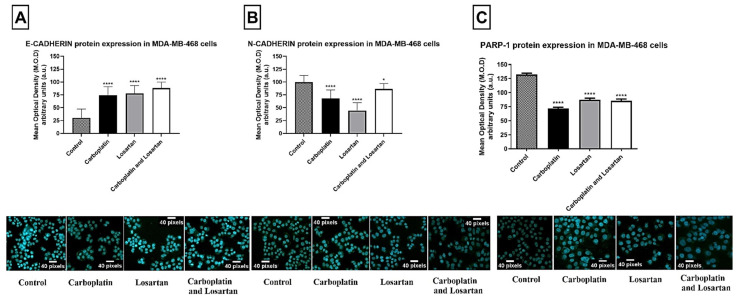
Protein expression of (**A**) E-cadherin, (**B**) N-cadherin, and (**C**) PARP-1 by immunofluorescence technique in human tumor cell line MDA-MB-468 comparing the groups treated with cells without treatment (control). Photomicrographs of immunofluorescence staining in magnification = ×100. Antibodies (green) and nuclei DAPI (blue). Each column represents the mean ± standard error of triplicate experiments. (*) *p* < 0.05 and (****) *p* < 0.0001 Significant value in the ANOVA test followed by Dunnett’s Multiple Comparison Test.

We assessed E-cadherin, N-cadherin, and PARP-1 protein expression in mammary tumor cell lines MDA-MB-468 and CF41 by immunofluorescence. After treatments, the cells were imaged by confocal microscopy and subsequent immunofluorescence-labelling of E-cadherin, N-cadherin, and PARP-1. For cells of the human mammary tumor cell line MDA-MB-468, there was a statistically significant increase in E-cadherin protein expression in all groups of treatment compared to the control group (*p* < 0.0001; Figure 1A). Otherwise, there was a decrease in N-cadherin and PARP-1 protein expression after treatment with carboplatin, losartan, or both in combination when compared to the control group (without treatment) (*p* < 0.0001; Figure 1B,C).

**Figure 2:**
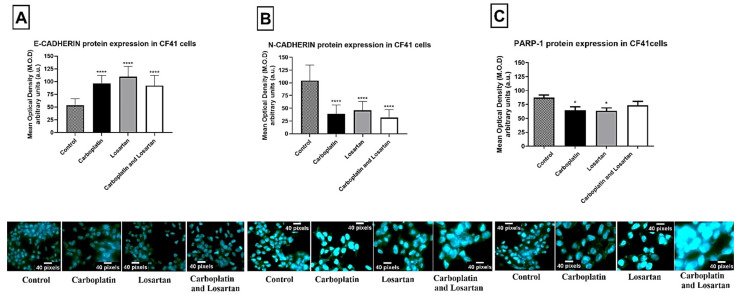
Protein expression of (**A**) E-cadherin, (**B**) N-cadherin, and (**C**) PARP-1 by immunofluorescence technique in canine mammary tumor cell line CF41 comparing the groups treated with cells without treatment (control). Photomicrographs of immunofluorescence staining in magnification = ×100. Antibodies (green) and nuclei DAPI (blue). Each column represents the mean ± standard error of triplicate experiments. (*) *p* < 0.05 and (****) *p* < 0.0001 Significant value in the ANOVA test followed by Dunnett’s Multiple Comparison Test.

Likewise, for canine mammary tumor cell line CF41.Mg, there was a statistically significant increase in E-cadherin (*p* < 0.0001; Figure 2A) protein expression and decrease in N-cadherin (*p* < 0.0001; Figure 2B), and PARP-1 (*p* < 0.0001; Figure 2C) after treatment with carboplatin, losartan, and with both in combination compared to the control group (without treatment).

### 3.2. Modulation of E-CADHERIN, N-CADHERIN, and PARP-1 Gene Expression after Losartan and Carboplatin Combined to the Losartan Treatment

**Figure 3:**
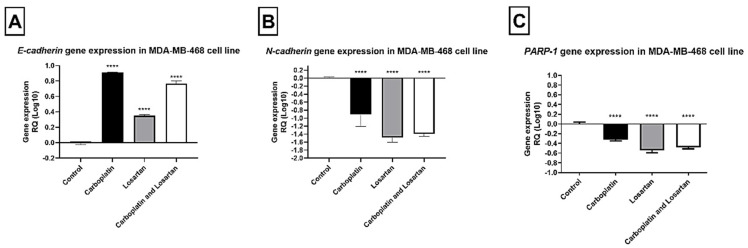
Gene expression of (**A***)* E-cadherin, (**B**) N-cadherin, and (**C**) PARP-1 by qRT-PCR in human tumor cell line MDA-MB-468 comparing the groups treated with cells without treatment (control). Each column represents the mean ± standard error of triplicate experiments. (****) *p* < 0.0001 Significant value in the ANOVA test followed by Dunnett’s Multiple Comparison Test.

The relative expression value of each gene of interest in this study was determined with the support of DataAssist v3.0 software, by the method of quantification in relation to the average of the endogenous control. The samples were tested in triplicate and, in all experiments, there was a negative control. For the human mammary tumor cell line MDA-MB-468, *E*-CADHERIN gene expression increased after all treatments (*p* < 0.05; Figure 3A). However, N-CADHERIN and PARP-1 gene expression decreased significantly after treatment with carboplatin, losartan, or both in combination compared to the control group (*p* < 0.05; Figure 3B,C).

**Figure 4:**
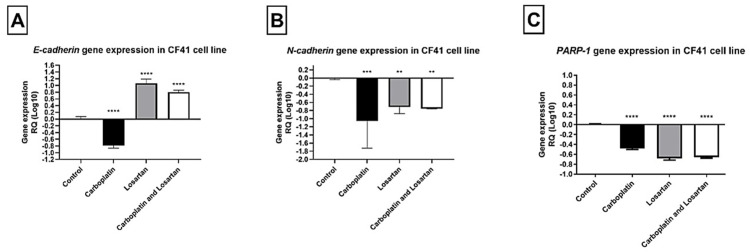
Gene expression of (**A**) E-cadherin, (**B**) N-cadherin, and (**C**) PARP-1 by qRT-PCR in canine mammary tumor cell line CF41comparing the groups treated with cells without treatment (control). Each column represents the mean ± standard error of triplicate experiments. (**) *p* < 0.01; (***) *p* < 0.001 and (****) *p* < 0.0001 Significant value in the ANOVA test followed by Dunnett’s Multiple Comparison Test.

In the same way, for canine mammary tumor cell line CF41, E-CADHERIN gene expression increased after treatment with carboplatin, losartan, or both in combination (*p* < 0.05; Figure 4A), while N-CADHERIN and PARP-1 gene decreased significantly after treatment with carboplatin, losartan, or carboplatin in combination with losartan compared to the control group (*p* < 0.05; Figure 4B,C).

### 3.3. AGTR-1 Gene Edition by CRISPR/Cas9 Decrease VEGF Protein and Gene Expression Modulating the Angiogenesis Process

**Figure 5:**
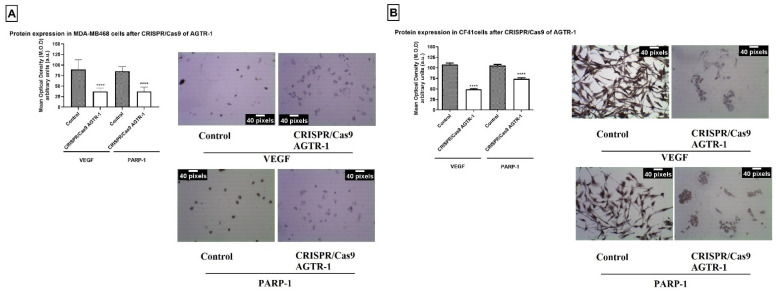
Protein expression of VEGF and PARP-1 after AGTR-1 gene edition by CRISPR/Cas9 by immunocytochemistry in (**A**) human tumor cell line MDA-MB-468 and (**B**) CF41 comparing to the group without treatment (control). The brown color represents the marking of the analyzed antibodies. Each column represents the mean ± standard error of triplicate experiments. (****) *p* < 0.0001 Significant value in the ANOVA test followed by Dunnett’s Multiple Comparison Test.

**Figure 6:**
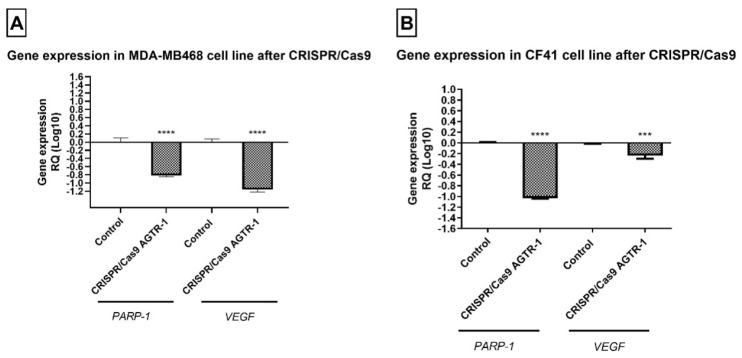
Gene expression of VEGF and PARP-1 by qRT-PCR in (**A**) human tumor cell line MDA-MB-468 and (**B**) CF41 comparing the groups treated with cells without treatment (control). Each column represents the mean ± standard error of triplicate experiments. (***) *p* < 0.001 and (****) *p* < 0.0001 Significant value in the ANOVA test followed by Dunnett’s Multiple Comparison Test.

To analyze the influence of AGTR-1 in angiogenesis process, the protein and gene expression of VEGF was analyzed by immunocytochemistry and PCR real time, respectively. The results showed that after the gene edition of AGTR-1 by CRISPR/Cas 9 there was a significantly decrease in VEGF and PARP-1 protein (*p* < 0.05; Figure 5) and gene (*p* < 0.05; Figure 6) expression in both cell lines. PARP is a marker for the cell’s DNA repair process, which is normally increased in expression in cancer cells. Our results showed a significant decrease in protein and gene expression of PARP-1 that demonstrates the role of AGTR-1 in control the DNA repair process.

### 3.4. Diminution of Cell Invasion after Treatment with Carboplatin, Losartan, and AGTR-1 Gene Edition by CRISPR/Cas9

**Figure 7:**
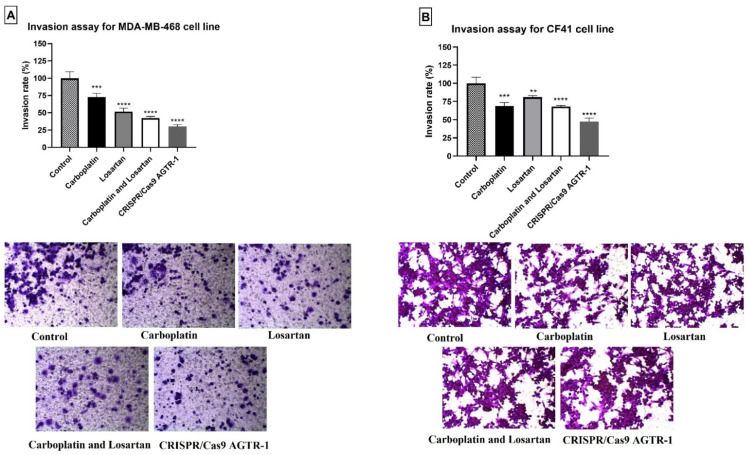
Invasion rate of cells for (**A**) human mammary tumor cell line MDA-MB-468 and (**B**) canine mammary tumor cell line CF41 comparing the groups treated with the control group (without treatment). (**) *p* < 0.01; (***) *p* < 0.0001 and (****) *p* < 0.0001 Significant value in the ANOVA test followed by the Bonferroni Multiple Comparison Test.

For the invasion assay, the rate was calculated using the average of the number of treated cells that migrated the matrigel membrane, divided by the average of the control cells that migrated the membrane. For the MDA-MB468 cell line, a statistically significant reduction of 27% in the invasion rate was observed when the cells were treated with carboplatin (72.69% ± 3.169), 48% when treated with losartan (51.73% ± 2.901), and 57% when combined losartan with carboplatin (42.49% ± 1.324), all treatments compared to the control group (100% ± 5.312). Furthermore, the gene edition of AGTR-1 by CRISPR/Cas9 (30.35% ± 1.641) significantly decrease the cell invasion capacity in 70% compared to the control group (*p* < 0.05; Figure 7A). For the CF41 canine mammary tumor cell line there was a statistically significant decrease in invasion rate after carboplatin (68.80 ± 2.715), losartan (80.77 ± 1.323), or both treatments in combination (67.95 ± 0.9432) compared to the control group (100.0 ± 4.796). After AGTR-1 gene edition by CRISPR/Cas9 technique (47.35% ± 2.696) there was a significant decrease in cell invasion rate compared to the control group (*p* < 0.05; Figure 7B).

## 4. Discussion

Angiogenesis is defined as the formation of new blood vessels from pre-existing vessels and is an essential process for the proliferation and viability of tumor cells. During cancer progression, angiogenesis initiates after a local imbalance between pro-angiogenic and anti-angiogenic factors, which leads to the recruitment of a new vascular supply to deliver essential nutrients and oxygen to the proliferating neoplastic cells. This process is promoted by the overexpression of pro-angiogenic factors, such as VEGF [23]. Activation of angiogenesis can lead to the EMT process, which is a biological program during which epithelial cells lose their cell identity and acquire a mesenchymal phenotype [24]. EMT is commonly observed during body development, wound healing, and tissue fibrosis [25]. However, this process can be developed by neoplastic cells and is often associated with resistance to apoptosis, tissue invasion, and resistance to treatment, which are characteristic of tumor stem cells [26].

Chemotherapy is still one of the most used modalities in cancer treatment. A variety of chemical agents, such as alkylating compounds and antimetabolites, have been used in cancer therapy to suppress the progression and invasion of neoplastic cells, affecting different cell characteristics. Recently, platinum-based drugs have attracted much attention due to their effectiveness in targeting different signaling pathways and mechanisms that control cancer progression. These compounds have shown great potential in the treatment of several types of cancer, including brain tumors, lung cancer, breast cancer, bladder cancer, prostate cancer, oral cancer, and neck cancer [27].

Our results demonstrated that losartan and carboplatin decreased the protein and gene expression of N-cadherin and increased E-cadherin in both cell lines. E-cadherin is one of the best-studied members of the cadherins superfamily and is a very important tumor suppressor gene, as downregulation of E-cadherin is often observed in malignant epithelial tumors. Furthermore, it is believed that a decrease in E-cadherin drives to the EMT mechanism, leading to an increase in tumor cell invasion and migration. On the other hand, several studies revealed that the upregulation of E-cadherin may be associated with a lower migratory capacity of tumor cells and greater sensitivity to cell death, which can be attributed to the inhibition of the EMT mechanism [28].

N-cadherin is normally absent or expressed at low levels in normal epithelial cells; altered expression of N-cadherin in epithelial tumor cells is a well-documented feature of malignant epithelial neoplasms such as breast, prostate, urothelial and pancreatic cancer, and is associated with disease progression. Likewise, upregulation of N-cadherin expression is a feature of melanoma progression. Although altered expression of N-cadherin in epithelial tissues is not considered to be oncogenic or growth-promoting in solid tumors, increased expression of N-cadherin is largely associated with tumor aggressiveness. In fact, many studies have demonstrated a significant correlation between high levels of N-cadherin in solid epithelial and non-epithelial tumors and clinicopathological features, such as increased localized tumor invasion and distant metastasis, and worse prognosis [29]. Although much interest has been generated in the last decade in relation to EMT as a potential therapeutic target, the development of new specific drugs against EMT or EMT-related signaling pathways constitutes a huge challenge in oncology and, at the moment, there are only a few based studies. in treatments that directly target EMT [30].

Angiotensin II type 1 receptor blockers (ARBs), including losartan, are commonly used to treat hypertension. Zhao et al. (2019) [31] used losartan to block angiotensin II signaling in ovarian cancer models. The authors did not observe direct proliferative or growth inhibitory effects in vitro after treatment with losartan, as well as not verifying the effects in vivo. However, the authors found that treatment with losartan increased the effectiveness of paclitaxel, facilitating drug delivery.

Losartan binds to AGTR-1, a component of the renin-angiotensin system (RAS), which classically regulates cardiovascular homeostasis. The AGTR-1 has shown potential to stimulate cell growth, migration, or invasion, and to promote angiogenesis, inflammation, and immunity. Studies have shown that the inhibition of AGTR-1 by antagonists such as losartan can suppress angiogenesis, contributing to the suppression of tumor growth and metastases [13].

Our results demonstrate that the AGTR-1 gene edition was able to decrease the VEGF and PARP-1 protein and gene expression. Furthermore, a decrease in cell invasiveness was observed after treatment with carboplatin, losartan, or AGTR-1 knockdown by CRISPR/Cas9 technique. Poly-ADP-ribose (PARP) is a nuclear protein that binds to DNA responsible for repair process which can be initiate by chemotherapy, leading to the resistance [32,33]. The increase in PARP-1 expression is associated with the overexpression of AGTR-1 and the treatment with the angiotensin II type 1 receptor antagonist, losartan, is capable to regulate the EMT process by decreasing TGFB expression that is an important regulator of metastasis and survival in patients with tumors [34].

In the literature, studies with mice have also demonstrated anti-tumor activity by losartan [34,35]. Retrospective studies of clinical data from patients being treated for hypertension demonstrated that the use of losartan provided better results also for the treatment of pancreatic, breast, or lung cancer [36]. In the pre-clinical rodent studies, losartan’s anti-tumor activity was attributed to anti-angiogenic or anti-TGF-β signaling effects; furthermore, there was evidence that losartan decreases the VEGF expression by regulating the angiogenesis process [37,38]. Despite the data in the literature, the mechanisms by which losartan acts to control tumor progression are not completely understood and have not yet been demonstrated in the canine species.

The limitations of the study are related to the fact that the work was performed in vitro, in human and canine breast cancer cell lines. Thus, it is necessary that the results obtained in vitro are validated in vivo, such as using xenographic models. However, the results shown in this study, especially in the CF41 canine cell line, are interesting and unprecedented, which may encourage further studies in this direction.

Taken together, the results have shown that losartan and the gene edition of AGTR-1 by CRISPR/Cas9 technique were able to decrease the angiogenesis process and consequently modulate the EMT process. Moreover, these treatments were able to control cancer invasion. It is important to note that no results with losartan in canine mammary tumors were found in the literature. The action of losartan in controlling tumor progression must be widely investigated as new target in BC and CM treatment to increase the chances of survival in these patients.

## Figures and Tables

**Table 1:** Cell density and transfection complex reagents.

	Reagent	96 Wells	24 Wells	6 Wells
**Cell density**	-	8000–18,000 cells	40,000–90,000 cells	250,000–450,000 cells
**Tube 1**	Opti-MEM™ I Medium	5 μL	25 μL	125 μL
	Cas 9 protein	250 ng (1.5 pmol)	1250 ng (7.5 pmol)	6250 ng (37.5 pmol)
	sgRNA	50 ng (1.5 pmol)	240 ng (7.5 pmol)	1200 ng (37.5 pmol)
	Lipofectamine™ Cas9 Plus™ Reagent	0.5 μL	2.5 μL	12.5 μL
**Tube 2**	Opti-MEM™ I Medium	5 μL	25 μL	125 μL
	Lipofectamine™ CRISPRMAX™ Reagent	0.3 μL	1.5 μL	7.5 μL

## Data Availability

The data presented in this study are available on request from the corresponding author. The data are not publicly available due to all data analyzed are available in the article.
